# Tango*: constrained synthesis planning using chemically informed value functions

**DOI:** 10.1039/d5dd00130g

**Published:** 2025-08-11

**Authors:** Daniel Armstrong, Zlatko Jončev, Jeff Guo, Philippe Schwaller

**Affiliations:** a Ecole Polytechnique Fédérale de Lausanne (EPFL) Lausanne Switzerland daniel.armstrong@epfl.ch philippe.schwaller@epfl.ch; b National Centre of Competence in Research (NCCR) Catalysis Switzerland

## Abstract

Computer-aided synthesis planning (CASP) has made significant strides in generating retrosynthetic pathways for simple molecules in a non-constrained fashion. Recent work has introduced specialized bidirectional search algorithms to find synthesis pathways that incorporate pre-selected starting materials, tackling a specific formulation of the starting material-constrained problem. In this work, we introduce a simple guided search—Tango*-which allows solving the starting material-constrained synthesis planning problem using an existing unidirectional search algorithm, Retro*. We show that by optimising a single hyperparameter, Tango* outperforms existing methods in terms of efficiency and solve rate. We also highlight the effectiveness of our computed node cost function in steering synthesis pathways.

## Abstract 2

TANGO* enables starting material constrained synthesis planning using computed molecular similarity to guide retrosynthetic search toward specific starting materials, achieving superior performance to neural network approaches.

## Graphical abstract

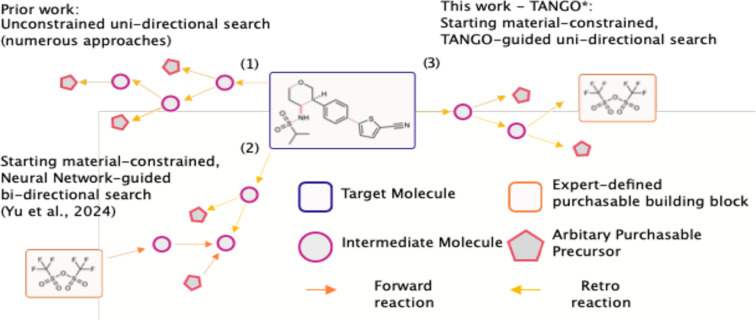

## 1 Introduction

Synthesis planning, where chemists design routes of chemical reactions to synthesise a complex molecule from simple or purchasable building blocks, is a key task in synthetic chemistry. The process used for this, retrosynthetic analysis, involves recursively performing reversed reactions, where a bond is broken to simplify a molecule into two or more component precursors.^1,2^ Originally proposed by Corey in 1969, Computer-Assisted Synthesis Planning (CASP) aims to automate this process.^3^ Since the seminal patent mining work of Lowe, which provided a large dataset of machine-readable chemical reactions, the CASP field has expanded significantly, with a plethora of approaches developed.^4–10^ CASP systems typically have two primary components: a single-step retrosynthesis model, which decomposes a molecule into simpler precursors, and a search algorithm that explores the search graph constructed from outputs of the single-step model.^6,8,11–14^ The iterative application of single-step models and exploration of the generated search space typically continues until a molecule is “solved,” which is specified as having all leaf nodes belonging to a predefined set of purchasable building blocks. This approach of finding a path to any available precursor differs substantially from the approach expert chemists may take, where chemists can plan a synthesis with numerous constraints in mind, such as avoiding certain reactions and solvents, or starting from a specific precursor, known as a “structure-goal”.^2^ By starting from a building block containing a key structural motif, the overall molecular complexity gain in a synthesis route can be lowered, a technique called “semi-synthesis”.^15,16^ There is also considerable interest in repurposing waste compounds into useful products, a technique called “waste valorisation”.^17–19^

**Fig. 1:**
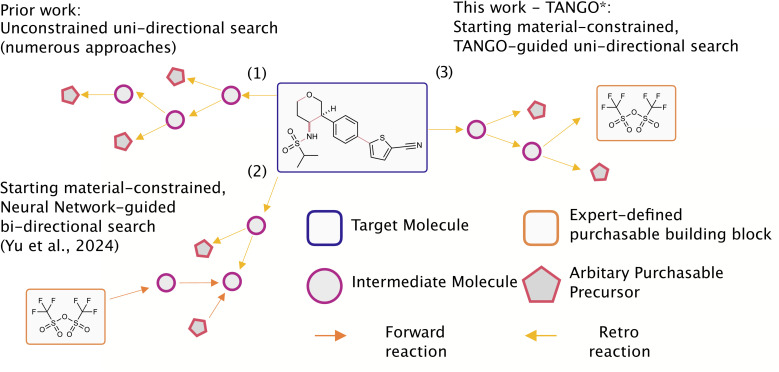
Comparison of existing constrained synthesis planning methods with Tango*. The approaches shown include: (1) unconstrained synthesis planning, (2) starting material constrained synthesis planning through bidirectional (F2F or F2E) search introduced by Yu *et al.*, and (3) our work, Tango*, which implements starting material constrained synthesis planning using computed cost function guided unidirectional search.

While designing constrained and steerable chemical synthesis is a daily practice in synthetic chemistry, it has received little attention in the CASP literature, with existing algorithms simply seeking to find any “valid” pathway to purchasable molecules (Fig. 1).

Recently, several approaches for starting material constrained synthesis planning have been proposed with promising results.^20–22^ Existing solutions either rely on rule-based approaches or require complex systems with several interacting parts. In this work, we show that a general-purpose and data-driven retrosynthesis system can be adapted to starting material constrained synthesis planning by the addition of a computed node cost function. Our contribution is as follows:

1. We use a computed node cost function, TANimoto Group Overlap (TANGO), to guide the retrosynthetic search process towards enforced blocks. In this work, these blocks are limited to starting materials but could include key intermediates or molecular substructures.

2. We show that by integrating TANGO into an existing general-purpose search algorithm, we can tackle the constrained synthesis planning problem with comparable or superior results to existing, specialised methods.

3. We further show that the TANGO node cost function can serve as a drop-in replacement for neural synthetic distance networks in existing specialised starting material constrained synthesis planning tools introduced by Yu *et al.*^22^.

4. We compare the outputs of existing retrosynthetic value functions with the outputs of the TANGO node cost function and present a plausible explanation for the improved performance over existing starting material constrained synthesis planning tools.

## 2 Related work

Computer-Assisted Synthesis Planning (CASP) tools typically formulate synthesis planning as a tree search, with each step corresponding to disconnecting a molecule into precursors through a “retro” chemical reaction. Two primary approaches are used for selecting retro reactions. Firstly, template-based methods extract chemical graph transformations from a corpus and train a neural network to select a transformation given an input.^6,11^ Template-free methods frame single-step retrosynthesis as a conditional language generation problem, with molecules encoded as SMILES strings or as a graph-edit prediction task.^10,12,23,24^ Additionally, models that leverage graph features for direct generation have been developed.^9,25^ Significant focus has been placed on how to use single-step models in multi-step synthetic planning. Initial approaches used hand-curated rules, while more recent methods use neural-network guided graph exploration, such as Monte Carlo Tree Search (MCTS) or AND-OR graph search methods.^8,11,13,14,26,27^ A key development was driven by Chen *et al.*,^8^ who proposed an A-star-like algorithm guided by a neural network that estimates the cost to synthesise a molecule from any arbitrary purchasable building block.^8^ More novel methods have utilised self-play and experience-based learning to improve navigation of the search space.^28–31^ While single-step model performance continues to improve, this has not always been translated into the real-world performance of multi-step CASP systems.^12,32–34^

### 2.1 Constrained single-step retrosynthetic models

In recent years, there has been increased focus on introducing constraints into single-step retrosynthesis models with specific goals. Toniato *et al.* utilised reaction class tokens to steer the output of single-step retrosynthetic transformers towards specific reaction classes.^35^ Following a similar approach, Thakkar *et al.* introduced “disconnection prompts” to guide single-step models to break specific bonds.^36^ In the multi-step planning domain, Westerlund *et al.* proposed a disconnection-aware transformer to encourage the breaking of bonds and allow the freezing of bonds during the search process, discarding any reaction that violates the frozen bond constraint.^37^ Interestingly, such bond constraints do not appear to impede the search process, indicating that simple, chemically informed rules can be powerful in data-driven retrosynthesis techniques.

### 2.2 Starting material constrained synthesis planning

Despite its potential use in waste valorisation and semi-synthesis, constrained synthesis planning has received limited attention in the literature. This approach imposes an additional constraint by focusing on the utilisation of specific starting materials. The LHASA program included such rules; however, they relied on expert-designed rules, limiting scalability.^20^ GRASP utilised reinforcement learning to develop a goal-driven synthesis planning tool that can target either arbitrary products or specific starting materials.^21^ Finally, some work has been done on starting material limited synthesisable molecular design, where the available set of building blocks is made substantially smaller than average, with the study showing that reducing the number of available building blocks from 17.4 million (Zinc) to 5955 (Led3), a decrease of approximately 3000-fold, results in only a 12% reduction in synthesis planning success rate when accepting synthesis routes that are, on average, two reaction steps longer.^38^ It is worth noting that in that work, a generative model proposes molecules satisfying the synthesisability metric. This does not directly transfer to the traditional CASP setting because the model has complete freedom (within the synthesisability constraint) to generate molecules. By contrast, our problem setting is that the target molecule is given. Recent state-of-the-art work proposed a bidirectional search algorithm, Double-Ended Synthesis Planning (DESP), which uses both forward- and retro-expansion models, guided by a value network that estimates the cost of synthesising molecule m2 specifically from molecule m1.^22^ DESP employs two search techniques, Frontier-to-Frontier (F2F) which compares all the nodes of each frontier with one another and Frontier-to-End (F2E) which compares the Retro* expansion frontier with the target starting material. Constrained synthesis planning has also emerged as a target in synthesisable molecular design. Guo *et al.* introduced a method for the *de novo* generation of synthesisable molecules using enforced building blocks in the synthesis pathway.^39^ To date, all starting material constrained synthesis planning tools have relied on specialised architectures, reinforcement learning, or expert-defined rules. In this work, we show instead that the problem can be approached with a simple cheminformatics calculation.
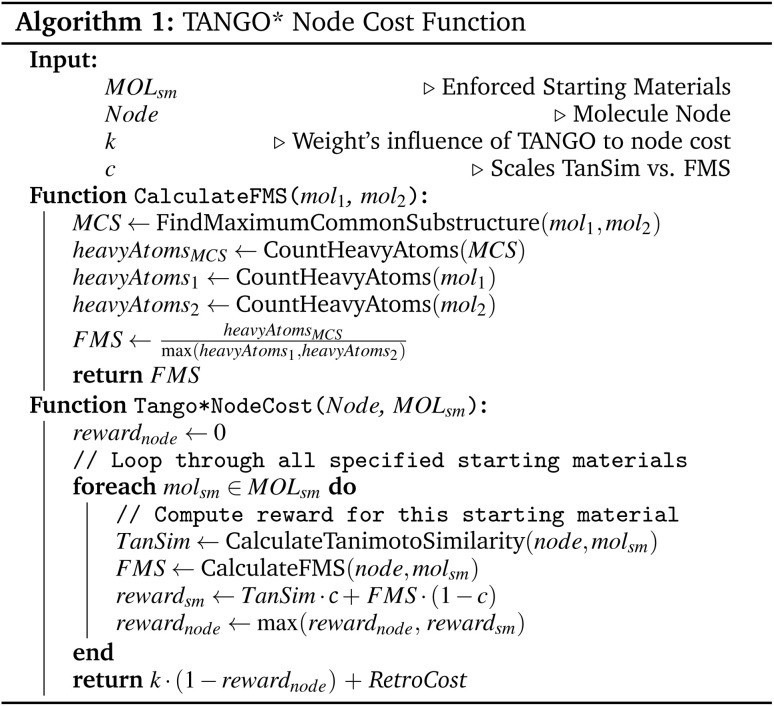


## 3 Methods

### 3.1 Datasets

In choosing datasets for analysis, we aim for continuity with prior work in starting material constrained synthesis planning and use the datasets introduced by Yu *et al.*^22^. This includes the common USPTO-190 dataset introduced by Chen *et al.*,^8^ which is a set of 190 challenging target molecules extracted from USPTO-Full. Additionally, we use the datasets introduced by Yu *et al.*,^22^ namely Pistachio Reachable and Pistachio Hard. The sets of target, starting material pairs are extracted for a set of commercial building blocks; we use canonical SMILES strings provided in the set of 23 million molecules from eMolecules used by Chen *et al.*^8^ and Yu *et al.*^22^. Specifically, we use the same sets of target, starting material pairs extracted in the DESP paper, which are extracted by finding the longest path from target to leaf node then picking the leaf node with the most heavy atoms. It is important to note that this experimental setup, also used by Yu *et al.*, means that the search is guided towards a building block that is already known to be part of a successful synthesis route for the target molecule. Therefore, the task is to recover a known-good synthetic connection within a pre-defined solution space, rather than discovering pathways to arbitrary starting materials. As we are using previously trained models with undefined Pistachio based training sets, we avoid analysing the algorithms on PaRoutes due to concerns about data leakage.

### 3.2 Machine learning models

To avoid variance due to subtle differences in data pre-processing techniques and to ensure a meaningful comparison, we use the Retro* value network and single-step retrosynthesis model provided by in previous work.^22,33^ Further details on model training can be provided in the Appendix A.4 of the DESP paper.^22^

### 3.3 Search algorithms

All algorithms are some variant of the best-first type introduced in Retro* by Chen *et al.*^8^. More details on this algorithm are provided in the SI. The Retro* + D and Tango* are based off this search algorithm with varying node cost functions. All algorithms use the baseline Retro* value network, which estimates the number of reactions required to synthesise molecule m1 from any commercially available starting material. The value will be referred to as Synthetic Distance for the rest of the manuscript. This cost is a positive real number, which is assigned to the code. Retro* + D utilises the same concept as the Retro* value network, but adapted to the starting material constrained setting, instead estimating the synthetic distance between a molecule and any starting material, it estimates the synthetic distance between a molecule and a predefined starting material. The Retro* cost is then subtracted from this to determine the overall node cost. Tango* replaces the D value network with the TANGO node cost function in Algorithm 1. For the Tango-F2F and Tango-F2E we replace the neural starting material guidance function in the original DESP methods with the TANGO node cost. Further details in the DESP paper.^22^

### 3.4 Tango* node cost function

The TANGO* node cost function uses a weighted sum of Tanimoto Similarity and Fuzzy Matching Substructure (FMS). The weighted FMS score is calculated by taking the number of heavy atoms in the maximum common substructure between two molecules, dividing by the number of heavy atoms in the precursor molecule, and multiplying by a weighting factor. This is formalised in 1.

### 3.5 Metrics

While there remains a lack of a clearly agreed-upon “gold standard” for reporting the quality of synthetic routes generated by CASP systems, a few metrics are commonly used. The Solve Rate in traditional CASP systems refers to the fraction of molecules for which there exists a synthetic pathway where all leaf nodes are in a set of purchasable building blocks. In this work, we used a modification of the solve rate metric introduced by Yu *et al.*,^22^ which we refer to as the constrained solve rate. This measures the fraction of target molecules for which a valid synthetic pathway exists, incorporating a predefined starting material (sm*), within an expansion budget E. This ensures that:

• The target molecule is synthesised.

• All reactants are either purchasable or can be derived from exclusively purchasable starting materials.

• The predefined starting material (sm*) is incorporated into the synthetic route.

• All requirements are met within the specified expansion budget.

The expansion budget which refers to the maximum allowed calls to the single step retrosynthesis model during the search process. The average route length refers to the number of reactions in the longest path from target to leaf node in the solved synthetic route, which is commonly used in the CASP literature as a proxy for route quality, as longer routes indicate verbosity in the solution. To ensure consistency across search algorithms, we only compare routes which all methods solved. In addition to reporting metrics regarding the outputs of Tango*, we aim to evaluate how efficient Tango* is at navigating the retrosynthetic search space and assess the effect of computational overhead on the system. We consider two measures for assessing the computational cost of our methods: average number of expansions (*N̄*) per target molecule, and average wall clock time per target molecule. The first provides a measure of how efficient our node cost function is at guiding the search towards a solution, while wall clock time combines this efficiency with the computational overheads of both the search algorithm and node cost functions. This is important as certain methods, particularly DESP-F2F, require substantially more calls to the node cost function per node expansion than unidirectional search methods.

### 3.6 Hyperparameter optimisation

We use a hyperparameter *k* to balance the starting material guidance of TANGO with the general guidance of the Retro* value network. To evaluate the ability of our method to generalise from simpler to more complex molecules, we choose the Pistachio Reachable dataset for hyperparameter tuning. We find a value of *k* = 25 optimises both constrained solve rate and average number of expansions. We employ an additional parameter, *c*, to specify the ratio of FMS to Tanimoto Similarity, with *c* defining the FMS weight. Through empirical testing, we determine the optimal value to be *c* = 0.3. In the results section, we will refer to Tango with *c* = 0.0 as Tango(1, 0) and Tango with *c* = 0.3 as Tango(0.7, 0.3).

## 4 Experiments

Our experiments are structured to answer the following questions:

1. Can a non-neural network computed node cost function be used to adapt general-purpose synthesis planning tools to the constrained setting?

2. Can such a system outperform existing specialised models for starting material-constrained planning?

3. Can the cost function additionally provide improvements to existing bidirectional search methods?

4. As the TANGO function is empirically computed as opposed to estimated, does TANGO generalise from simple to harder datasets more effectively?

**Fig. 2:**
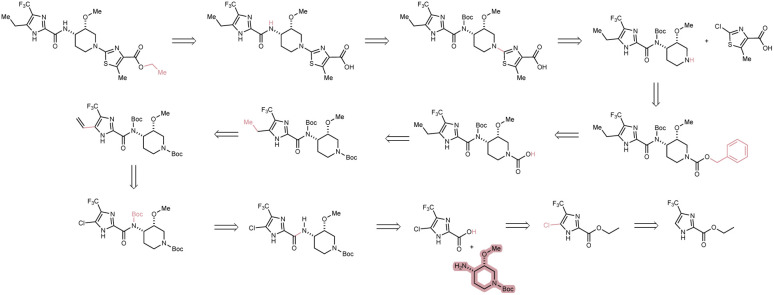
Here we demonstrate a meaningful 12-step route generated by our method on a (target, starting material) pair not solved by the best performing DESP^22^ method. Constrained starting material highlighted in red; bonds/atoms disconnected shown in red.

The primary results are displayed in Table 1. Tango(1, 0)* demonstrates improvements in the starting material-constrained setting and consistently outperforms the neural network-enhanced Retro* (referred to as Retro* + D) across all benchmarks and expansion limits while displaying greater algorithmic efficiency, as measured by the average number of expansions (*N̄*). In addition, Tango(1, 0)* achieves higher or comparable solve rates to both DESP methods across all three datasets, doing so with a strictly lower average number of expansion calls, clearly demonstrating the utility of the TANGO reward to navigate the retrosynthetic action space (see for Fig. 2 an example). We find that the value of *k*, optimised on Pistachio Reachable, shows strong generalisation performance to the more challenging datasets, with Tango* being the best-performing method across all iteration levels on USPTO-190.

**Table 1:** Summary comparison between baseline methods and Tango* across the three benchmarks. Baseline results for Retro*, GRASP, Retro* + D and the DESP methods are taken from Yu *et al.*^22^. Solve rate is the fraction of (target, starting material) pairs solved within the expansion limit. Tango DESP methods use a (1, 0) weighting of Tanimoto Similarity to FMS. Best overall results are in bold and best unidirectional search results are underlined

Algorithm	USPTO-190				Pistachio reachable				Pistachio hard			
	Constrained solve rate (%) ↑			*N̄* ↓	Constrained solve rate (%) ↑			*N̄*↓	Constrained solve rate (%) ↑			*N̄* ↓
	Expansion budget				Expansion budget				Expansion budget			
	100	300	500		50	100	300		100	300	500	
Random	4.2	4.7	4.7	479	16.0	26.7	40.7	325	6.0	12.0	13.0	452
BFS	12.1	20.0	24.2	413	48.7	57.3	74.0	169	16.0	26.0	29.0	390
MCTS	20.5	32.1	35.3	364	52.0	72.7	85.3	111	27.0	31.0	32.0	361
Retro*	25.8	33.2	35.8	351	70.7	78.0	92.7	73	32.0	35.0	37.0	342
GRASP	15.3	21.1	23.7	410	46.7	51.3	66.7	198	14.0	22.0	29.0	402
Retro* + D	27.4	32.6	37.4	348	77.3	87.3	96.0	49	31.0	40.0	42.0	323
DESP-F2E	30.0	35.3	39.5	340	84.0	90.0	96.0	41	35.0	44.0	50.0	300
DESP-F2F	29.5	34.2	39.5	336	84.5	88.9	97.3	38	39.0	45.0	48.0	293

**Ours**												
Tango(1, 0)*	**36.3**	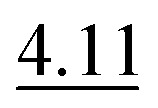	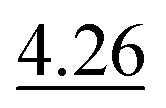	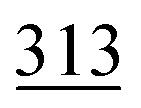	84.5	90.6	97.3	32	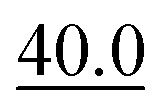	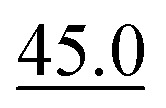	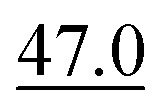	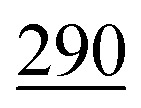
Tango(0.7, 0.3)*	35.7	40.5	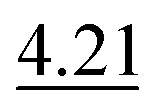	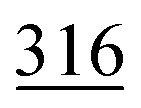	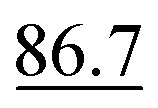	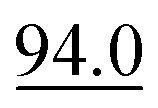	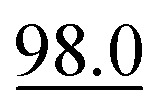	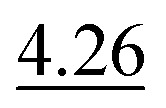 29	39.0	44.0	46.0	295
Tango-F2E	33.1	40.0	41.5	317	88.7	92.0	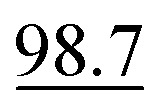	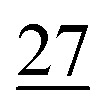	39.0	45.0	49.0	290
Tango-F2F	33.2	**45.3**	**53.7**	**291**	**91.3**	**95.3**	**99.3**	**18**	**47.0**	**59.0**	**63.0**	**231**

We perform an ablation of the Tanimoto Similarity: FMS Similarity weighting in the TANGO cost function, which we refer to as Tango(0.7, 0.3). We find that although the incorporation of FMS into the cost function improves the solve rate and reduces expansion calls for Pistachio Reachable, such results do not carry over to the more challenging datasets. We hypothesise that Tanimoto Similarity offers greater granularity for guidance than FMS, enabling higher performance on more complex datasets.

**Fig. 3:**
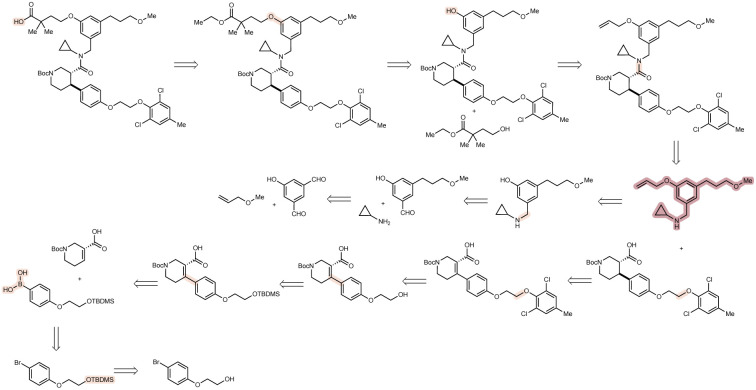
Here we demonstrate a feasible 10-step route generated by Tango-DESP-F2F on a (target, starting material) pair not solved by the neural guided DESP-F2F method. Constrained starting material is highlighted in red; bonds/atoms disconnected are shown in red.

To examine the general applicability of the TANGO cost in guiding various search algorithms, we explore its integration into the recently proposed bidirectional search methods, DESP-F2F and DESP-F2E.^22^ This integration is achieved by replacing the pairwise synthetic distance network, *D*, with the TANGO cost function. The hyperparameters *k* and *c* are set to the same values as Tango(1, 0). Our findings show that the addition of TANGO reward generally leads to a substantial increase in the solve rate for both DESP methods, while also reducing the average number of expansions and route length. Particularly noteworthy is the impressive performance of Tango-F2F at high expansion budgets, where it achieves a 99.3% solve rate on Pistachio Reachable and improves accuracy by approximately 25% compared to the next best method on the more challenging USPTO-190 and Pistachio Hard datasets. An example of a successful route is shown in Fig. 3. We note that as the added bidirectional search of DESP outperforms Retro* + D, TANGO-DESP methods should be expected to outperform Tango*.

### 4.1 Wall clock time and route length ablations

As both DESP and Tango* introduce computational overhead that may add ambiguity to the number of expansion calls compared to the computational resources required, we report the wall clock time. Tango* consistently achieves a lower wall clock time than alternative starting material-constrained methods (Table 2). Finally, we investigate the average number of reactions per solved route for each method. Tango* achieves shorter route lengths than all existing methods on USPTO-190, but only matches existing methods on other datasets. The strongest results in terms of route length come from the combination of TANGO with the bidirectional search algorithms F2E and F2F, one of them displaying the shortest routes for all of the datasets. This result is revealing; existing methods use a neural network directly trained to predict synthetic distance, yet it fails to provide significantly stronger guidance towards shorter synthetic routes than a simple molecular similarity measure. This leads to the question: just how effective are such neural networks at estimating the synthetic distance of a node, and how reliable is this estimation at test time?.

**Table 2:** Inference time and mean solved route length for the evaluated methods. Route length comparisons are made on the routes solved by all methods

Algorithm	USPTO-190		Pistachio reachable		Pistachio hard	
	Route length (61 routes)	Wall clock time (s)	Route length (114 routes)	Wall clock time (s)	Route length (36 routes)	Wall clock time (s)
Retro*	5.30	58.1	4.64	10.2	4.67	56.3
Retro* + D	5.56	64.1	4.67	8.3	4.67	55.2
DESP-F2E	5.13	66.5	4.51	8.6	4.56	56.3
DESP-F2F	5.51	109.4	4.46	8.2	4.44	61.8
Tango(1, 0)*	5.06	**55.8**	4.56	5.8	4.67	**47.5**
Tango-F2E	**4.44**	75.3	4.24	6.5	**4.29**	54.2
Tango-F2F	5.06	146.4	**4.04**	**4.9**	4.40	72.8

### 4.2 Why does Tango* work?

Despite access to a starting material constrained node cost function (one with access to information from ground truth routes at test time), Retro* + D does not show a substantial increase in performance compared to Retro*. In contrast, Tango*’s incorporation of a privileged node cost function provides significant performance improvements.

We hypothesize that, as a computed cost function, the TANGO cost function should be relatively invariant to the molecular inputs and maintain strong performance at test time. Let 
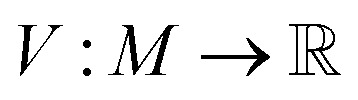
 be a node cost function where *M* is the space of molecules. For node cost to effectively guide retrosynthetic search, the function should ideally provide consistent and discriminative estimates across synthetic pathways. While perfect monotonicity (where *V*(*m*_*i*_) > *V*(*m*_*i*+1_) for all *i* ∈ {1, …, *n* − 1} along a synthetic path *m*_1_, …, *m*_*n*_ from root to starting material) would be desirable, we recognize that this may be challenging to achieve in practice due to factors such as “tactical combinations,” where synthetic complexity may temporarily increase during retrosynthesis to enable major complexity-reducing reactions Gajewska *et al.*^40^.

More fundamentally, we expect effective cost functions to demonstrate two key properties: consistency—providing reliable estimates with low variance for molecules at similar synthetic distances—and granularity—offering discriminative power to distinguish between molecules at different synthetic distances. We further expect these properties to be more pronounced for routes that are solved by a method compared to routes not solved, as this indicates that the node cost function can effectively guide search tree exploration.

To systematically evaluate these hypotheses about TANGO's effectiveness and empirically assess the relative strength of different guidance functions, we analyse their behavior on ground truth synthetic routes in the test set. Using the USPTO-190 dataset, we extract the linear synthetic path from root molecule *m*_r_ to expert-defined starting material *m*_s_. We define synthetic distance *d*(*m*_1_, *m*_2_) as the minimum number of reactions required to synthesise *m*_1_ from *m*_2_. For each molecule *m*_i_ in this path, we calculate the following values:

• *d*(*m*_i_, *m*_s_): ground truth synthetic distance.

• D(*m*_i_, *m*_s_): Neural network estimation of synthetic distance.

• T(*m*_*i*_, *m*_*s*_): TANGO cost molecular similarity.

To isolate the cost function, we fix the search algorithm, in this case focusing on the Retro* algorithm with either TANGO or D as a starting material-guided cost function. We then take the 4 sets of routes that are solved and not solved by Tango* and Retro* + D. We plot the corresponding starting material constrained cost function, TANGO and D respectively, as costs for each node in the ground truth synthetic routes.

**Fig. 4:**
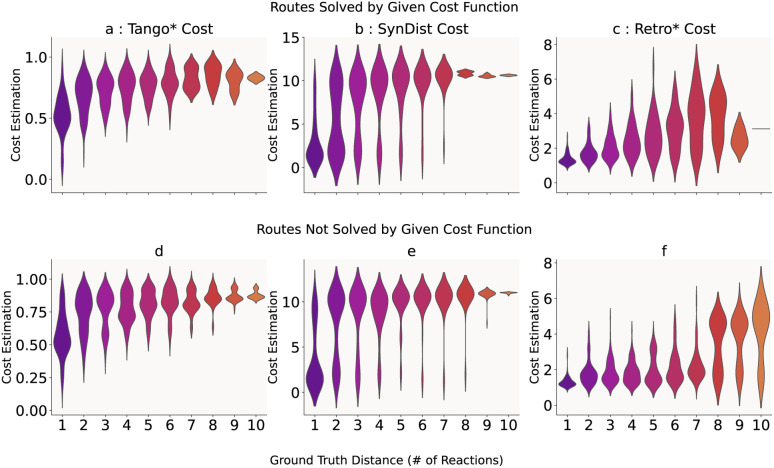
A comparison of node cost estimates for USPTO-190 routes solved and not solved by Retro* search using the corresponding cost function. (a) Tango cost for routes solved by Tango*, (b) SynDist cost for routes solved by Retro* + D, (c) Retro* cost function estimates for routes solved by Retro*, (d) Unweighted Tango cost for routes not solved by Tango*, (e) SynDist cost for routes not solved by Retro* + D, and (f) Retro* cost for routes not solved by Retro*. The values on the top right of the plots indicate the Kendall's Tau coefficient of monotonicity. The colours demark different ground truth synthetic distances.

We show the results of this experiment in Fig. 4 and Table 3. The neural network estimated synthetic distance (b) and (e) displays an unexpected bimodal distribution, with peaks at low and high estimates. It displays consistently high absolute error and is unable to provide a granular estimate of synthetic distance. For routes that Retro* + D solves, the mean synthetic distance estimate shows high variability (mean distribution overlap = 1.107, Table 3) and inconsistency (mean CV = 0.334), with the estimation function struggling to provide reliable distance estimates across the synthetic pathway. This is exacerbated for routes that Retro* + D does not solve, where the synthetic distance estimation shows even greater distributional overlap (2.555) and variability (CV = 0.310), consistently varying around the fixed training value of 10 regardless of the actual ground truth distance.

**Table 3:** Consistency and granularity metrics for different cost functions on solved and unsolved routes. Lower mean distribution overlap and mean coefficient of variation indicate better consistency and granularity, while Spearman's indicates monotonicity. We note that these metrics are computed on the appropriated weighted TANGO node cost used in the implemented algorithm, while the violin plots in 4 displays 1 − TANGO

Node cost function	Route status	Spearman's *ρ*	Mean distribution overlap	Mean coefficient of variation
TANGO	Solved	0.553	**0.478**	**0.141**
	Not solved	0.504	0.584	**0.135**
SynDist (D)	Solved	0.645	1.107	0.334
	Not solved	0.662	2.555	0.310
Retro*	Solved	0.653	1.359	0.321
	Not solved	0.524	1.822	0.366

We note that this fits with the training strategy of D described in Yu *et al.*,^22^ which augments the training set with synthetic “negative samples” of (target, starting material) pairs. These samples are generated by selecting two molecules that are disconnected in the directed graphs formed by linking reactants and products in USPTO, and are assigned a fixed “distance” value of 10.

In comparison, the computed TANGO cost function (a) and (d) exhibits substantially improved consistency and granularity (Table 3). For routes solved by Tango*, the cost function demonstrates low distributional overlap between adjacent synthetic distances (0.478), indicating clear separation between cost estimates at different pathway positions. The function also shows a high consistency within distance groups (CV = 0.141), suggesting more reliable cost estimation. Importantly, TANGO maintains reasonable performance even for unsolved routes, with modest increases in overlap (0.584) and variability (CV = 0.135), indicating the function's robustness across different route types.

The Spearman correlation values (ranging from 0.504 to 0.662 across all conditions, Table 3) suggest moderate but consistent rank-order relationships between synthetic distance and cost estimates for TANGO, while the neural distance function shows similar correlation values but with much higher variance and overlap, indicating less reliable guidance for search algorithms.

We hypothesize that this improved consistency and granularity—rather than perfect monotonicity—enables TANGO cost-guided search algorithms to achieve substantially improved solve rates compared to neural network-guided methods. The key advantage appears to be TANGO's ability to provide discriminative and consistent cost estimates that effectively separate molecules at different synthetic distances, while neural approaches suffer from high variance and poor granularity that hampers effective search guidance.

### 4.3 Case study: synthesis of useful compounds from renewable/waste feedstock

**Fig. 5:**
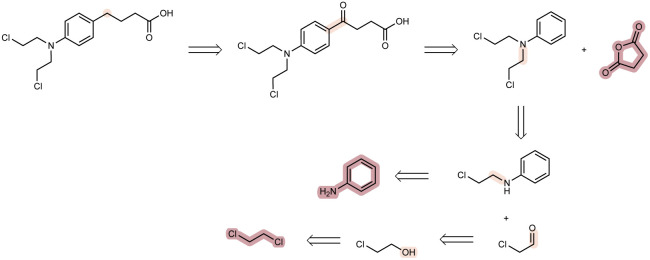
Here we show a feasible synthesis route to the chemotherapy drug, Chlorambucil, a WHO essential medicine, synthesised entirely from renewable or industrial waste feedstocks.

A key aim of starting-material-constrained synthesis planning is to enable the discovery of synthetic pathways to useful compounds from renewable or waste feedstocks. Previous evaluations relied on a database of 23 million chemical building blocks from the eMolecules database. We aim to demonstrate the effectiveness of our method, Tango*, in finding synthesis pathways to useful small molecules starting exclusively from renewable or waste feedstocks. For renewable building blocks, we use a set of 146 small molecules previously curated by Wołos *et al.*^17^. For useful compounds, we extract a set of 110 small molecules from a curation of the WHO list of essential medicines, previously developed by Gao *et al.*^41^. We conduct the search using the Tango(1, 0) set of hyperparameters previously described, but set the expansion budget to 1000 model calls. In Fig. 5, we present a strong route, discovered by Tango* but not Retro*, to the chemotherapy drug Chlorambucil, starting exclusively from renewable starting materials. We find all proposed reactions and the complete synthesis are directly reported in the literature.^42,43^

## 5 Conclusion

In this work, we introduce Tango*, a simple adaptation of the Retro* algorithm to the starting material-constrained setting without any model retraining. We demonstrate that our TANGO guided search method strictly outperforms the similar neural network-guided Retro* + D. Despite relying on single-ended search, Tango* either outperforms or matches the performance of specialised DESP models and search algorithms, providing routes that satisfy the specified goal for a greater number of compounds. Application of the TANGO node cost function to the DESP methods also yields substantial improvements, particularly to the F2F method, which achieves the strongest solve rate performance of all investigated systems. It proposes routes with a comparable length and does so with a lower number of expansion calls and reduced wall clock time.

We show that existing neural node cost functions fail to provide a granular and monotonic decrease in node cost throughout a retrosynthesis pathway, particularly struggling on more challenging routes. In contrast, the computed Tango* cost function displays better monotonicity and granularity on both solved and unsolved routes. This work indicates that there may be substantial room for improvement in developing novel guidance functions for retrosynthesis tools.

We anticipate that future developments in similar methods will unlock synthesis planning tools with diverse and flexible structure constraints, allowing expert chemists to specify key intermediates or predefined substructure goals at any position in the synthetic route.

## Author contributions

Daniel Armstrong: conceptualisation, methodology, software, investigation, writing – original draft, writing – review and editing, Zlatko Joncev: writing – review and editing, Jeff Guo: writing – review and editing and Philippe Schwaller: supervision, writing – review and editing.

## Conflicts of interest

The authors declare no conflicts of interest.

## Supplementary Material

DD-004-D5DD00130G-s001

## Data Availability

The data used in this study are sourced from the Double-Ended Synthesis Planning (DESP) paper, which is available on Figshare at https://figshare.com/articles/preprint/Pre-trained_models_and_data_for_Double-Ended_Synthesis_Planning_DESP/25956076. The source code for the Tangostar project can be found in the GitHub repository at https://github.com/schwallergroup/TangoStar – DOI (https://doi.org/10.5281/zenodo.16418756). All computational methods and data processing scripts are included in the repository to ensure reproducibility of the research results. This document provides additional details on the computational methods, algorithms, datasets, hyperparameter tuning and additional experiments that support the main findings of the publication. See DOI: https://doi.org/10.1039/d5dd00130g.
