# Supplementary material for: Tango*: constrained synthesis planning using chemically informed value functions
Source: Digit Discov. 2025 Aug 11;4(9):2570–8. doi: 10.1039/d5dd00130g (PMC12355204; doi:10.1039/d5dd00130g)
Supplement: DD-004-D5DD00130G-s001 [file DD-004-D5DD00130G-s001.pdf]

Supplementary Information (SI) for Digital Discovery.
This journal is © The Royal Society of Chemistry 2025

Supplementary Information – Tango*: Constrained synthesis planning using chemically informed value functions

A.1: Compute Details

Wall clock time comparisons were implemented on a GPU-enabled workstation with the following specifications.

- CPU: 12-core AMD Rysen 9 7900X
- RAM Memory : 64 GB
- GPU : NVIDIA A6000 48 GB

A.2: Retro* Algorithm

We note this algorithm description is largely taken from the DESP paper.

Retro* defines the following quantities:

- V_m: For a molecule m, V_m is an unconditional estimate of the minimum cost required to synthesise m. This estimate is provided by a neural network.
- $\mathrm{rn}(m|G)$: For a molecule m in the search graph G, the "reaction number" $\mathrm{rn}(m|G)$ represents the estimated minimal cost to synthesise m.
- $V_t(m|G)$: For a molecule m in the search graph G with the target molecule p^*, $V_t(m|G)$ denotes the estimated minimal cost to synthesise p^* starting from m.

Retro* operates through iterative phases of selection, expansion, and update. We follow the DESP implementation of Retro* as follows:

Selection:

Choose the molecule from the set of frontier nodes F that minimises the expected cost of synthesising the target p^* given the current search graph G:

$$m_{\text{select}} = \arg\min_{m \in F} V_t(m|G) \tag{1}$$

Expansion:

As detailed in Algorithm 2, apply a one-step retrosynthesis model to the selected node m_{select}, and add the resulting reactions and precursor molecules to the search graph G. Initialise each new molecule node with:

$$\mathrm{rn}(m|G) \leftarrow V_m$$

Update:

First, propagate the reaction number values upward to ancestor nodes. For a reaction node R, update its reaction number as the sum of its childrens' reaction numbers plus the cost of the reaction $c(R)$:

$$\mathrm{rn}(R|G) \leftarrow c(R) + \sum_{m \in \mathrm{ch}(R)} \mathrm{rn}(m|G) \tag{2}$$

For a molecule node m, update its reaction number to be the minimum reaction number among its child reactions:

$$\mathrm{rn}(m|G) \leftarrow \min_{R \in \mathrm{ch}(m)} \mathrm{rn}(R|G) \tag{3}$$

Next, propagate the values of $V_t(m|G)$ downward to descendant nodes. Starting from the target molecule p^*:

$$V_t(p^*|G) \leftarrow \mathrm{rn}(p^*|G) \tag{4}$$

For subsequent reaction nodes R, update the value as:

$$V_t(R|G) \leftarrow \mathrm{rn}(R|G) - \mathrm{rn}(\mathrm{pr}(R)|G) + V_t(\mathrm{pr}(R)|G) \tag{5}$$

Finally, for molecule nodes m that are not the target p^*:

$$V_t(m|G) \leftarrow \min_{R \in \mathrm{pr}(m)} V_t(R|G) \tag{6}$$

Here, $\mathrm{ch}(R)$ denotes the set of child molecules of reaction R, and $\mathrm{ch}(m)$ represents the set of child reactions of molecule m. Similarly, $\mathrm{pr}(R)$ denotes the parent molecule of reaction R, and $\mathrm{pr}(m)$ represents the set of parent reactions of molecule m.

This implementation ensures that at each iteration, the algorithm selects the most promising node to expand based on the estimated cost, propagates cost updates throughout the search graph, and efficiently guides the search towards the most cost-effective synthesis pathways.

A.3: Dataset Details

We provide details on the used datasets in Table 1. The Table is taken directly from the original paper **?**.

Table 1: Benchmark dataset summary. Avg. In-Dist. % is the mean percentage of reactions in each route within the top 50 suggestions of the retro model. Unique Rxn.% is the ratio of deduplicated reactions to total reactions across all routes. Avg. # Rxns. is the mean number of reactions in each route, and Avg. Depth is the mean number of reactions in the longest path of each route.

Dataset	# Routes	Avg. In-Dist. %	Unique Rxn. %	Avg. # Rxns.	Avg. Depth
USPTO-190	190	65.6	50.5	6.7	6.0
Pistachio Reachable	150	100	86.1	5.5	5.4
Pistachio Hard	100	59.9	95.2	7.5	7.2

A.4: TANGO Hyperparameter Screening

Here we provide a hyperparameter screen of TANGO weight and Tanimoto weight across the 3 Tango augmented methods, **Tango***, **TANGO DESP-F2E** and **TANGO DESP-F2F**. Due to computational resource limitations, this screen was conducted exclusively on Pistachio Reachable with a small expansion budget of 50. In general, a higher Tango Weight (¿ 15) tends to lead to a higher solve rate, consistent accross all methods.

A Tanimoto weight of 0.75 performs strongly for the **TANGO DESP-F2F Tango*** methods on the Pistachio Reachable test set, consistent with previous results.

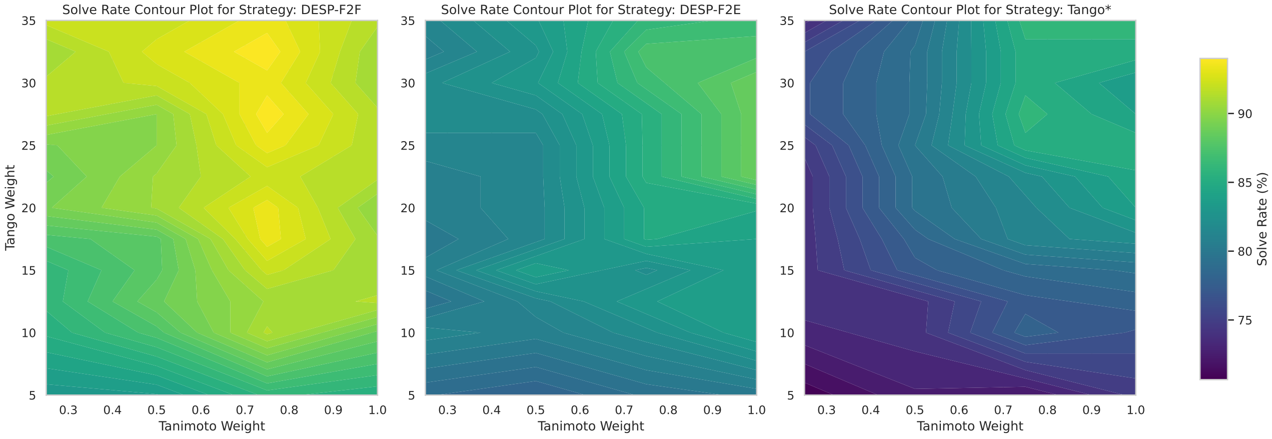

Figure 1: A hyper-parameter screen conducted on Pistachio Reachable with an expansion limit of 50.

A.5: Renewable synthesis of WHO Essential Medicines datasets

The sources of small molecule WHO Essential Medicines and renewable and waste compound feeds**??** are not machine-readable. We used a vision-enabled large language model (GPT-4o) to parse compound names from the Supplementary Information sections of the aforementioned papers.

A.6: Value function failiure points

In Figure 7, we analyze discrepancies between model-predicted number of synthesis steps (Retro* and SynthDist) and ground truth values across four synthetic transformations. In these examples, models overestimate synthetic complexity compared to ground truth, likely due to their focus on structural features rather than strategic synthetic planning. This is evident in: (a) ring-opening of tetrahydropyran introducing rotatable bonds of an alkyl chain, (b) TBDMS protection adding heavy atoms, (c) oxidation of primary alcohols changing atom connectivity and adding heavy atoms, and (d) lactone formation creating additional ring complexity. These cases demonstrate how the models may misinterpret strategic intermediates with increased synthetic complexity as being further away from commercially available building blocks while in reality, these intermediates help disconnections over multiple steps, suggesting potential areas for improvement in retrosynthetic prediction algorithms.

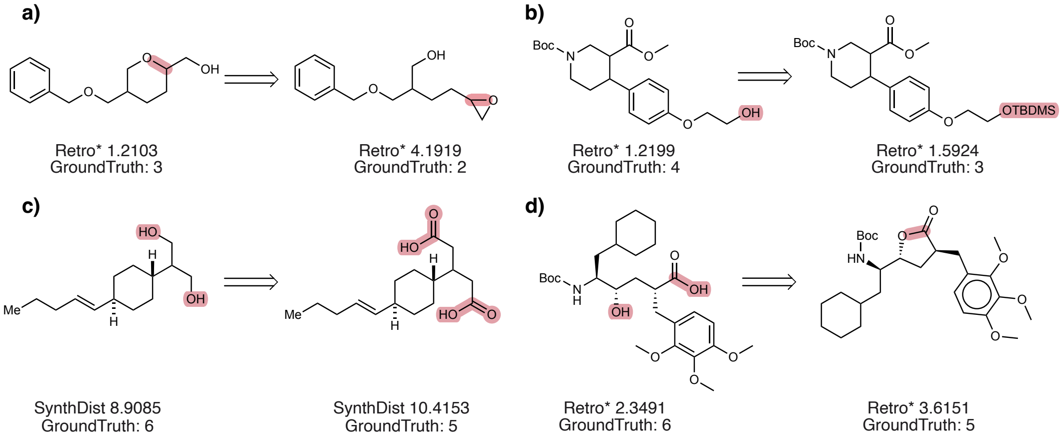

Figure 2: Failure points of value networks (Retro* and SynDist) compared with ground truth synthetic distance values. Bonds and atoms that are being modified are highlighted.

A.7 Algorithm evaluation in the unconstrained setting

Here we conduct an experiment to evaluate the impact of starting material guidance in the traditional, unconstrained CASP setup. We find that solve rates are strictly higher than the constrained setting but no significant differences between methods are found. We refrain from evaluating the DESP-F2F and F2E methods as the bottom up expansion makes them inseparable from the starting material constrained setting. Tango* is run with the same settings used in Table 1 of the main manuscript.

Table 2: Evaluation of starting material guided search on unconstrained synthesis planning.

Algorithm	USPTO-190				Pistachio Reachable				Pistachio Hard			
	Solve Rate (%) ↑			$\overline{N}$ ↓	Solve Rate (%) ↑			$\overline{N}$ ↓	Solve Rate (%) ↑			$\overline{N}$ ↓
	100	300	500		100	300	500		100	300	500	
Retro	47.9	57.4	64.7	158.1	98.0	99.3	99.3	11.4	63.0	74.0	78.0	110.3
Retro-SD	47.9	58.9	65.8	157.1	98.0	99.3	99.3	11.4	63.0	75.0	78.0	107.0
Tango(0.7, 0.3)*	47.9	57.9	65.8	157.0	98.0	99.3	99.3	11.4	63.0	75.0	78.0	109.1
